# Cartilage calcification of the ankle joint is associated with osteoarthritis in the general population

**DOI:** 10.1186/s12891-018-2094-7

**Published:** 2018-05-24

**Authors:** Jan Hubert, Lukas Weiser, Sandra Hischke, Annemarie Uhlig, Tim Rolvien, Tobias Schmidt, Sebastian Karl Butscheidt, Klaus Püschel, Wolfgang Lehmann, Frank Timo Beil, Thelonius Hawellek

**Affiliations:** 1 Department of Trauma Surgery, Orthopaedics and Plastic Surgery, University Medical Center Göttingen, Robert-Koch-Straße 40, 37075 Göttingen, Germany; 2 Department of Medical Biometry and Epidemiology, University Medical Center Hamburg-Eppendorf, Hamburg, Germany; 3 Department of Urology, University Medical Center Göttingen, Göttingen, Germany; 4 Department of Osteology and Biomechanics, University Medical Center Hamburg-Eppendorf, Hamburg, Germany; 5 Department of Legal Medicine, University Medical Center Hamburg-Eppendorf, Hamburg, Germany

**Keywords:** Cartilage calcification, Calcium crystals, Chondrocalcinosis, Ankle joint, Osteoarthritis, Cartilage

## Abstract

**Background:**

Cartilage calcification (CC) is associated with osteoarthritis (OA) in weight-bearing joints, such as the hip and the knee. However, little is known about the impact of CC and degeneration on other weight-bearing joints, especially as it relates to the occurrence of OA in the ankles. The goal of this study is to analyse the prevalence of ankle joint cartilage calcification (AJ CC) and to determine its correlation with factors such as histological OA grade, age and BMI in the general population.

**Methods:**

CC of the distal tibia and talus in 160 ankle joints obtained from 80 donors (mean age 62.4 years, 34 females, 46 males) was qualitatively and quantitatively analysed using high-resolution digital contact radiography (DCR). Correlations with factors, such as the joint’s histological OA grade (OARSI score), donor’s age and BMI, were investigated.

**Results:**

The prevalence of AJ CC was 51.3% (95% CI [0.40, 0.63]), independent of gender (*p* = 0.18) and/or the joint’s side (*p* = 0.82). CC of the distal tibia was detected in 35.0% (28/80) (95% CI [0.25, 0.47]) and talar CC in 47.5% (38/80) (95% CI [0.36, 0.59]) of all cases. Significant correlations were noted between the mean amount of tibial and talar CC (*r* = 0.59, *p* = 0.002), as well as between the mean amount of CC observed in one ankle joint with that of the contralateral side (*r* = 0.52, *p* = 0.02). Furthermore, although the amount of AJ CC observed in the distal tibia and talus correlated with the histological OA-grade of the joint (*r* = 0.70, *p* < 0.001 and *r* = 0.72, *p* < 0.001, respectively), no such correlation was seen in the general population with relation to age (*p* = 0.32 and *p* = 0.49) or BMI (*p* = 0.51 and *p* = 0.87).

**Conclusion:**

The prevalence of AJ CC in the general population is much higher than expected. The relationship between the amount of AJ CC and OA, independent of the donors’ age and BMI, indicates that CC may play a causative role in the development of OA in ankles.

## Background

Osteoarthritis (OA) is a major health problem that affects about 15% of the global population [1]. While OA in weight-bearing hip and knee joints is relatively common, in the ankle joint (AJ) it affects only 1% of the population [2]. It is often hypothesized that the development of AJ OA is mostly related to previous trauma [3, 4]. Valderrabano et al. reported a high prevalence of post-traumatic AJ OA (in 78% of cases) [4], while, other studies have shown a considerably lower prevalence (only 14%) [5]. The real impact of trauma on the development of AJ OA is yet to be fully understood, and the ability to accurately predict which patients will develop AJ OA in the future requires further investigations.

It is likely that the variations in individuals’ articular cartilage composition will play a role in the development of AJ OA. Eckstein et al. reported surprisingly high variability in the quantitative distribution of cartilage in the ankles of patients [6], whereas Quinn et al. found intraindividual variations in the cartilage cells and matrix morphologies of knees and ankle joints [7].

Another possible explanation for the development of AJ OA could be the occurrence of calcification within the hyaline articular cartilage, also known as chondrocalcinosis [8]. A high prevalence of cartilage calcification (CC) as well as a significant correlation between CC and OA has been reported in both weight-bearing hip and knee joints as well as in the first metatarsophalangeal joint (MTP-I joint) [9–13]. Furthermore, in vitro studies have shown that calcium phosphate crystals can alter cartilage tissue via biomechanical [14, 15] and pro-inflammatory biochemical processes [16–19], all of which can lead to degeneration of the affected joint.

The prevalence of ankle joint cartilage calcification (AJ CC) in the general population is reported at around 4.7%, and is based on only one cross-sectional study in which the occurrence of calcification on the talar surface was analysed macroscopically [20] and an association between CC and OA of the talus was reported. However, early signs of CC are only measurable in the nano- to micrometre ranges, thus raising the possibility of underestimation with conventional imaging techniques. In order to detect the onset of CC, high-resolution imaging techniques like digital contact radiography (DCR) are required [21]. Taking this into account, the precision of the previously reported cross-sectional study might be called into question [20].

Therefore, the primary goal of this study was to evaluate and quantify the prevalence of AJ CC using high-resolution DCR. Secondly, we examined the correlations between the observed CC with age, BMI and the histological grade of osteoarthritis.

## Methods

Both ankle joints (*n* = 160) of 80 donors were obtained from an unselected cohort who underwent autopsy at the Department for Legal Medicine, University Medical Center Hamburg-Eppendorf [22]. Only donors with bilaterally intact ankle joints with no signs of any other diseases (except for OA) were included in this study. Donors with a history of previous ankle surgery, tumours, infections and/or rheumatic diseases were excluded. The study was approved by the local Ethics Committee (PV 4570) and is in compliance with the Helsinki Declaration.

### Sample preparation

**Fig. 1:**
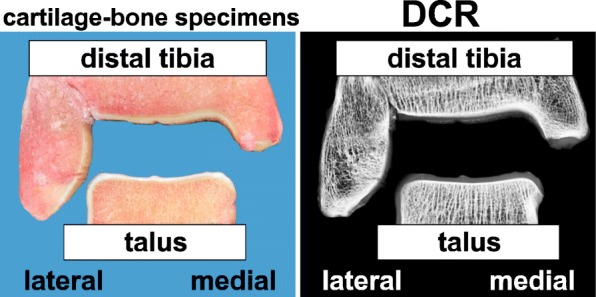
Examplary samples of the ankle joint showing standardized, 4 mm cartilage-bone specimens (cut along the coronal plane) of the distal tibia and talus, as well as the corresponding digital contact radiographs. Calcification was detectable as radiopaque spots in the cartilage’s matrix

Firstly, the whole ankle joint of the right and left limbs were extracted. Next, the soft tissue was carefully removed from the talus and the distal tibia along with the corresponding tibiofibular joint. For the calcification analysis, standardized 4 mm cartilage-bone specimens were cut in the coronal plane of the talus and the distal tibia along with the corresponding tibiofibular joint (Fig. 1).

### Digital contact radiography (DCR)

The prepared cartilage-bone specimens were then washed with physiological saline solution to remove residual bone debris before being subjected to standardized radiography (25 kV, 3.8 mAs, film focus distance of 8 cm) using a high-resolution digital radiography device (Faxitron X-Ray, Illinois, USA). Calcifications were detected as radiopaque spots within the cartilage matrix. Subsequently, the radiographs were qualitatively and quantitatively analysed using standard software (ImageJ 1.46, National Institutes of Health, Bethesda, USA) [9, 23]. The amount of calcification was determined as the percentage of the total area of the hyaline cartilage.

### Histology

**Fig. 2:**
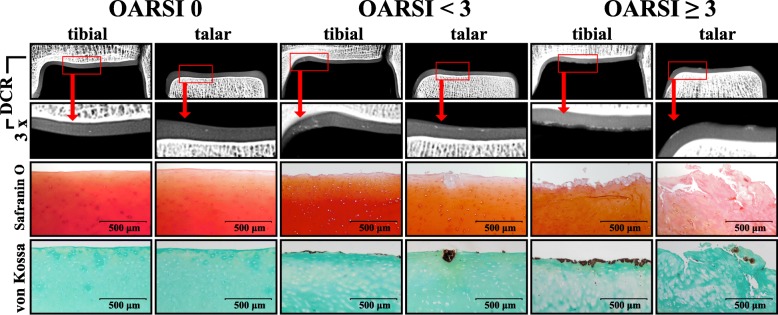
Representative DCR-images (original size and 3× magnification as shown in red boxes) of the cartilage-bone specimens taken from the distal tibia and talus of three donors with different OA grades (i.e. OARSI = 0, OARSI < 3 and OARSI ≥3). The corresponding histological images of the distal tibial and talar cartilage are presented. Safranin-O staining was used to evaluate the histological OA grade of the hyaline cartilage. Calcification was histochemically confirmed using von Kossa staining

The histological OA grade was evaluated for the talar and distal tibial cartilage (central load-bearing zone) of all ankle joints. Therefore, a sample of full thickness hyaline cartilage of the previously extracted cartilage-bone-specimen was cut to the subchondral bone plate. All cartilage samples were fixed in 4% PFA for 24 h before being dehydrated using 80% alcohol and embedded in paraffin. Four-μm sections of all samples were stained with 1% Safranin-O (Fig. 2) in order to evaluate the samples’ histological degeneration grade as it relates to the OARSI osteoarthritis cartilage histopathology assessment system (Grades 0 to 6) [24]. To confirm the occurrence of calcium phosphate deposition, von Kossa staining was performed.

### Statistical analysis

The biometric characteristics of donors are reported as mean values ±standard deviations. For descriptive analysis, mean CC values for each joint were used. Logarithmic transformation was performed for further evaluation. Fisher’s test was conducted to obtain categorical data, whereas side comparisons were evaluated using McNemar’s Exact test. Differences between the mean amount of distal tibial and talar CC were analysed using a linear mixed model. The model takes into account the values of a donor’s left and right ankle joints and uses them as random effects with compound symmetry covariance structure (as opposed to using the joint as a fixed effect). In addition, assumptions for the mixed model were checked using residual plots. To determine the association between continuous variables Pearson’s (r) or Spearman’s (r_s_) rank correlation coefficient was calculated. Partial correlation calculations were carried out using the respective parameters (CC, histological degeneration grade and age) adjusted to avoid spurious correlations. All statistical analyses were performed with Software R, Version 3.1.1. [25]. *P*-values of less than 0.05 were considered statistically significant.

## Results

The mean age of the study population was 62.4 years (SD ±17.7, range 23–95 years). Thirty-four of the donors were female, whereas 46 were male. Biometric characteristics of the study population are presented in Table 1.

**Table 1:** Biometric characteristics of the study population (*n* = 80)

Age [years]	62.4 ± 17.7
Male	59.2 ± 17.9
Female	66.6 ± 16.7
Height [cm]	
Male	177.5 ± 7.1
Female	161.7 ± 7.9
Body weight [kg]	
Male	81.9 ± 17.9
Female	72.3 ± 11.5
Body Mass Index [kg/m^2^]	25.4 ± 4.9

### Prevalence of cartilage calcification

In our study population, the prevalence of AJ CC was 51.3% (41/80) (95% CI [0.40, 0.63]). The left joint was affected in 37.5% (30/80) (95% CI [0.27, 0.49]) while the right joint in 40.0% (32/80) (95% CI [0.29, 0.52]) of all cases. No side showed signs of higher susceptibility to CC (*p* = 0.82). Bilateral CC was detected in 26.3% of donors (21/80). The prevalence of talar CC was 47.5% (38/80) (95% CI [0.36, 0.59]), whereas CC of the distal tibia was 35.0% (28/80) (95% CI [0.25, 0.47]). Bilateral talar CC was noted in 17.5% (14/80), while bilateral CC of the distal tibia was seen in only 8.8% (7/80) of all cases (Table 2).

**Table 2:** Prevalence of DCR-detectable cartilage calcification (*n* = 80)

	Ankle		Talus		Distal tibia	
	n	%	n	%	n	%
Total CC	41/80	51.3	38/80	47.5	28/80	35.0
Bilateral CC	21/80	26.3	14/80	17.5	7/80	8.8
Unilateral	20/80	25.0	24/80	30.0	21/80	26.3
Left CC	30/80	37.5	26/80	32.5	15/80	18.8
Right CC	32/80	40.0	26/80	32.5	20/80	25.0
Male	27/46	58.7	24/46	52.2	18/46	39.1
Female	14/34	41.2	14/34	41.2	10/34	29.4

### Gender

AJ CC was detected in 58.7% (27/46) (95% CI [0.43, 0.73]), talar CC in 52.2% (24/46) (95% CI [0.37, 0.67]) and distal tibial CC in 39.1% (18/46) (95% CI [0.25, 0.55]) of all male donors. In the female donor cohort AJ CC was observed in 41.2% (14/34) (95% CI [0.25, 0.59]), talar CC in 41.2% (14/34) (95% CI [0.25, 0.59]) and distal tibial CC in 29.4% (10/34) (95% CI [0.15, 0.47]) (Table 2). There were no significant differences regarding the prevalence of AJ CC (*p* = 0.18), talar CC (*p* = 0.37) or distal tibial CC (*p* = 0.48) for gender.

### Quantitative analysis of cartilage calcification

**Fig. 3:**
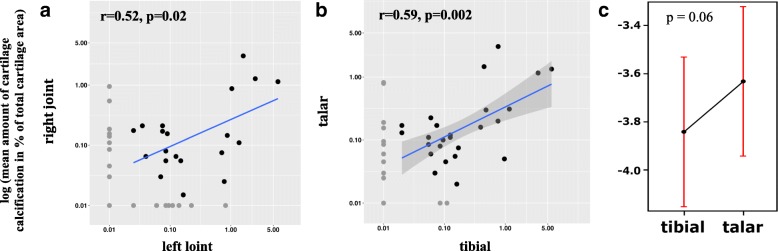
**a**, **b**. Logarithmic scatter plots (with blue orthogonal regression lines) showing significant correlations between the mean amount of CC in (**a**) the right and left ankle joints and (**b**) between the talar and distal tibial cartilage. Data points have been adjusted to avoid over-plotting. **c** The mean amount of calcification in the distal tibial and talar cartilage is depicted as an Effect Plot (logarithmic)

The mean amount of AJ CC was quantitated at 0.17% (SD ± 0.52, range: 0.00–3.55); left AJ CC 0.22% (SD ± 0.77, range: 0.00–5.97) and right AJ CC 0.13% (SD ± 0.41, range: 0.00–3.03). Significant correlations were noted between the two joints (*r* = 0.52, *p* = 0.02) (Fig. 3a).

The mean amount of talar CC was quantified at 0.15% (SD ± 0.43, range: 0.00–3.04) and distal tibia CC at 0.39% (SD ± 1.53, range: 0.00–11.53). Significant correlations were found between the mean amount of CC noted in the talar and distal tibial cartilage (*r* = 0.59, *p* = 0.002) (Fig. 3b), however, no quantitative differences could be detected (*p* = 0.06) (Fig. 3c).

### Cartilage degeneration (OARSI score)

The mean histological degeneration grade of the left/right distal tibia was 1.5 (SD ± 1.0, range: 0–5)/1.5 (SD ± 1.0, range: 0–6) and of the left/right talus 1.3 (SD ±1.1, range: 0–6)/1.6 (SD ± 1.1, range: 0–5). The distribution of the histological OA grade according to the OARSI score system (Grade 0–6) is presented in Table 3.

**Table 3:** Distribution of the histological OA-grade by OARSI (*n* = 80)

	Distal tibia				Talus			
	Left		Right		Left		Right	
OARSI	n	%	n	%	n	%	n	%
0	9	11.3	12	15.0	18	22.5	12	15.0
1	37	46.3	33	41.3	35	43.8	32	40.0
2	26	32.5	26	32.5	17	21.3	21	26.3
3	5	6.3	7	8.8	6	7.5	11	13.8
4	1	1.3	1	1.3	3	3.8	3	3.8
5	2	2.5	0	0.0	0	0.0	1	1.3
6	0	0.0	1	1.3	1	1.3	0	0.0

### Cartilage calcification and histological degeneration

#### Distal tibial cartilage

**Fig. 4:**
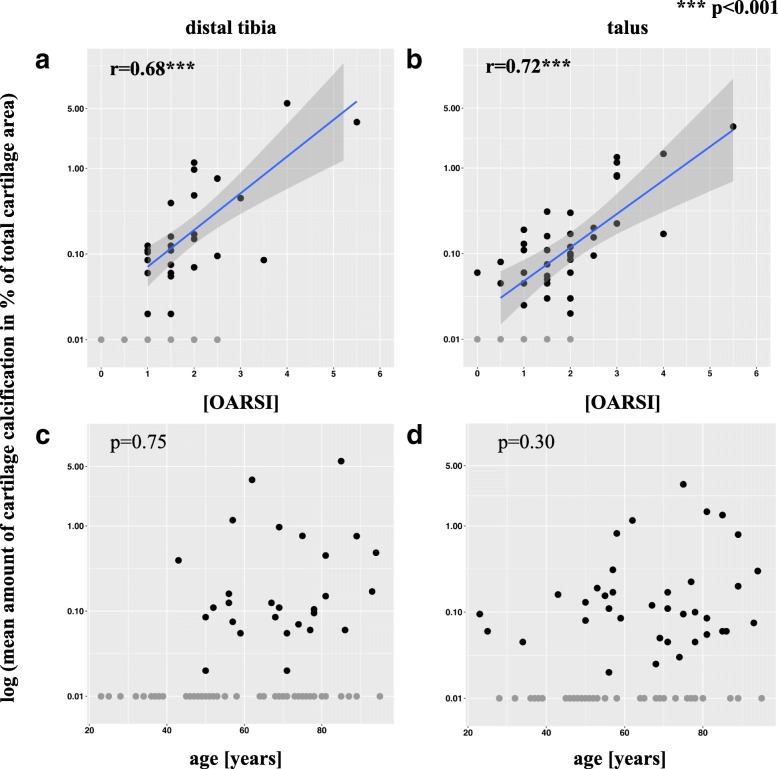
Logarithmic scatter plots (with blue orthogonal regression lines) showing correlations between the mean amount of CC and the histological degeneration grade (OARSI) of the distal tibial (**a**) of the talar cartilage (**b**) between the mean amount of CC and age for the distal tibia and (**c**) for the talus (**d**). Data points have been adjusted to avoid over-plotting

Distal tibial CC was detected in only 11.9% (17/143) of the cases that were classified as ‘mild cartilage damage’ (OARSI < 3). However, distal tibial CC was reported in 82.4% (14/17) of the cases with ‘severe cartilage degeneration’ (OARSI ≥3), (Table 4). Quantitative analysis revealed significant correlations between the mean amount of distal tibial CC and the histological degeneration, both without (*r* = 0.70, *p* < 0.001, 95% CI [0.44, 0.85]) and after an adjustment for age (*r* = 0.68, *p* < 0.001) (Fig. 4a). Conversely, there was no significant correlation between the amount of distal tibial CC and the histological degeneration of the talus (*r* = 0.23, *p* = 0.25).

**Table 4:** Distribution of joints with mild (OARSI < 3) and severe (OARSI ≥3) OA-grade with positive CC (*n* = 160)

	Distal tibia		Talus	
OARSI	n	%	n	%
< 3	17/143	11.9	29/135	21.5
≥ 3	14/17	82.4	23/25	92.0

#### Talar cartilage

Talar CC was detected in only 21.5% (29/135) of the cases with ‘mild cartilage damage’ (OARSI < 3) and in 92.0% (23/25) of the cases with ‘severe cartilage degeneration’ (OARSI ≥3) (Table 4). Overall, a significant correlation was observed between the amount of talar CC and the histological degeneration grade of the talus, both without (*r* = 0.72, *p* < 0.001, 95% CI [0.52, 0.85]) and after an adjustment for age was conducted (*r* = 0.72, *p* < 0.001) (Fig. 4b). Additionally, significant correlations were noted between the amount of talar CC and distal tibial degeneration (*r* = 0.61, *p* < 0.001, 95% CI [0.36, 0.78]).

### Cartilage calcification and age

#### Distal tibial cartilage

No correlation was observed between the amount of distal tibial CC and age without (*p* = 0.32) and after an adjustment for histological degeneration grade (*p* = 0.75) (Fig. 4c).

#### Talar cartilage

There was no correlation between the amount of talar CC and age without (*p* = 0.49) and after an adjustment for histological degeneration grade (*p* = 0.30) (Fig. 4d).

### Cartilage calcification and BMI

No correlation was detected between the amount of CC and the BMI of the donor for both the distal tibial (*p* = 0.51) and the talar cartilages (*p* = 0.87).

### Histological degeneration grade and age

Correlations were noted between the histological degeneration grade of the distal tibial/talar hyaline cartilage and age ((*r* = 0.28, *p* = 0.01, 95% CI [0.06, 0.47])/(*r* = 0.40, *p* < 0.001, 95% CI [0.20, 0.57])).

## Discussion

Independent of the donor’s gender and side, an unexpectedly high prevalence of AJ CC (51.3%) was found in this study. Intraindividual correlations existed between the amount of CC of the left with that of the right AJ, as well as between the distal tibial and the talar cartilage. These findings underline the systemic appearance of ankle-related CC. Furthermore, since calcification has already been found in intact AJ cartilage and the amount of CC correlated with the histological OA grade, we hypothesize that CC may play a causative role in the pathogenesis of AJ OA.

So far, the prevalence of ankle-related CC has been reported in only one cross-sectional study [20]. However, since calcium phosphate deposition is known to begin in the nano- to micrometre range, it is almost impossible to detect the early stages of calcification through macroscopic analysis or even using standard radiographic techniques. High-resolution imaging techniques such as Digital Contact Radiography (DCR) are therefore necessary for detection of the onset of CC [21]. Using DCR, we were able to establish that CC was prevalent in 51.3% of all cases; this was in stark contrast to previously published studies in which the prevalence of CC was a mere 4.7% [20]. Despite this, DCR has shown that AJ CC is relatively rare when compared to the prevalence of CC in other joints, such as the shoulder (98.9%) [26], the hip (96.6%) [11] or the knee (94.3–100%) [11, 23]. Nonetheless, the reason for the difference remains elusive. Interestingly, calcification is comparably prevalent in both the AJ and the MTP-I joint (48.1%) [12]. It has been theorized that weight-loading promotes pro-mineralization of the joints [27, 28], however, the results of our study contradict this point. Since the ankle generally bears enormous loads (many times greater than body weight), it stands to reason that the degree of calcification should be higher. Moreover, other studies have reported that calcification is more prevalent in non-weight bearing joints such as the shoulder [26] and has even been observed in non-weight-bearing parts of the knee cartilage [29]. Another factor to investigate is the impact of the donor’s BMI on CC (additional mechanical stress induced by increasing BMI). We could not find any association between the donor’s BMI and the mean amount of AJ CC. Given this, it can be assumed that mechanical load is not the predominant factor.

Our study highlighted significant correlations between the mean amount of CC in the left and right AJ, between the left and right distal tibia and talus, as well as correlations between the mean amount of calcification in the distal tibial and talar cartilage. These results underline the theory that the development of calcification is systemic [11, 26, 30].

Another interesting observation was the correlation between CC and the donor’s histological OA grade. CC was detected in donors with histologically intact or almost intact hyaline cartilage (i.e. an OARSI grade <  3) in 12% and in 22% of the distal tibial and talar cartilage respectively. Given this, it can be indicated that CC is already present in the joint before the histological OA is even measurable, and might occur before the OA process initiates. Similar observations of spontaneous OA development have been found in two animal models [31, 32], wherein calcification was detectable before cartilage degeneration occurred.

In comparison with other joints [9–13, 26, 30, 33], there is also a clear association between CC and OA in the ankle. In our study, CC in the distal tibia was detectable in 82% of the donors with severe OA (i.e. an OARSI grade ≥ 3), whereas talar CC was detected in 92%. Moreover, our quantitative analysis demonstrated that the mean amount of calcification in the distal tibial, as well as in the talar cartilage correlated with the histological degeneration grade. Muehlemann et al. also described an association between the prevalence of CC and macroscopic talar degeneration, even though no quantitative analysis was conducted for their study [20]. Taken together, there seems to be evidence that CC plays a crucial role in the development of AJ OA.

No correlation was found between the mean amount of AJ CC and the donors’ age. This is in line with previously published results for other joints, including the shoulder [26], the hip/knee [11] and the MTP-I joint [12]. In contrast, Mitsuyama et al. [23] observed significant correlations between the mean amount of CC in the knee and age of the general population. However, since no adjustment for the donor’s OA grade was conducted, it is conceivable that this association between CC and donor age might have been a spurious correlation, which would disappear once an adjustment for OA grade would be performed.

Certainly, there are some limitations to this study. There were no information about the donor lifestyle, activity and medical history, in particular ankle complaints. Even though the standardized cartilage-bone specimens of the distal tibia and talus used in this study were representative, they reflected only a small proportion of the ankle articulating surface. Lastly, the calcium-phosphate composition of DCR-detected CC was not thoroughly characterized in our study since such analyses require the use of specific diagnostic methods, e.g. FTIR spectroscopy [34] or X-Ray diffractometry [35], and were not specifically in the scope of our study. Nevertheless, none of these limitations is likely to influence the study’s findings and conclusions.

## Conclusion

DCR analysis revealed that the prevalence of ankle-related cartilage calcification is much higher than previously considered in general population. Even though it is independent of the donor’s age and/or BMI, calcification seems to occur in histologically intact ankle cartilage and is linked with the joint’s histological OA grade. These insights indicate that hyaline CC is an early, age-independent element and a possible causative factor in the development of ankle-related osteoarthritis. However, the exact pathophysiological role of CC in osteoarthritis and its subsequent importance in the disease’s molecular mechanisms are yet to be identified and investigated.

## Abbreviations

AJ: Ankle joint
CC: Cartilage calcification
DCR: Digital-contact radiography
OA: Osteoarthritis
OARSI: Osteoarthritis cartilage histopathology assessment system
